# Empirical Treatments for Male Infertility: A Focus on Lifestyle Modifications and Medicines

**DOI:** 10.3390/diseases12090209

**Published:** 2024-09-11

**Authors:** Aris Kaltsas, Athanasios Zachariou, Fotios Dimitriadis, Michael Chrisofos, Nikolaos Sofikitis

**Affiliations:** 1 Third Department of Urology, Attikon University Hospital, School of Medicine, National and Kapodistrian University of Athens, 12462 Athens, Greece; ares-kaltsas@hotmail.com (A.K.); mxchris@yahoo.com (M.C.); 2 Department of Urology, Faculty of Medicine, School of Health Sciences, University of Ioannina, 45110 Ioannina, Greece; zahariou@otenet.gr; 3 Department of Urology, Faculty of Medicine, School of Health Sciences, Aristotle University of Thessaloniki, 54124 Thessaloniki, Greece; helabio@yahoo.gr

**Keywords:** male infertility, lifestyle modifications, medical treatments, sperm quality, reproductive health

## Abstract

Background/Objectives: Male infertility is a prevalent issue impacting numerous couples worldwide. This review aims to evaluate the effectiveness of empirical therapies for male infertility, focusing on both lifestyle modifications and medical treatments. This study provides a comprehensive overview of interventions aimed at improving male fertility outcomes. Methods: A thorough review of the existing literature was conducted, encompassing studies on lifestyle changes such as dietary changes, smoking cessation, alcohol moderation, and exercise. Additionally, medical treatments including selective estrogen receptor modulators, gonadotropins, aromatase inhibitors, phosphodiesterase-5 inhibitors, antioxidants, dopamine agonists, kallikrein, indomethacin, low-dose corticosteroids, alpha-blockers, and nitric oxide donors were evaluated. The study population included males diagnosed with infertility, focusing on various underlying causes. Results: Lifestyle modifications were found to have a positive impact on sperm quality. Evidence shows that a healthy diet, smoking cessation, moderate alcohol consumption, and regular exercise improve fertility outcomes. Medical treatments demonstrated significant improvements in sperm production and quality. Selective estrogen receptor modulators and gonadotropins enhanced sperm parameters. Aromatase inhibitors and phosphodiesterase-5 inhibitors specifically improved sperm motility and increased pregnancy rates. Antioxidants, such as vitamins E and C and coenzyme Q10, reduced oxidative stress and enhanced sperm counts, motility, and morphology. Dopamine agonists, particularly cabergoline, normalized prolactin levels and improved fertility outcomes. Kallikrein therapy improved sperm parameters and increased pregnancy rates. Indomethacin treatment was associated with increased sperm concentrations and motility. Low-dose corticosteroids and alpha-blockers showed variable results, and nitric oxide donors like L-arginine enhanced sperm counts and motility. Conclusions: Empirical therapies, including lifestyle modifications and medical treatments, significantly enhance sperm quality and reproductive potential. These integrated approaches are essential in improving fertility outcomes in males. However, further extensive randomized trials are necessary to definitively establish the most effective treatments.

## 1. Introduction

Male factor infertility accounts for approximately half of all infertility cases and affects around 15% of couples [1]. While various factors can contribute to male infertility, in some cases, no identifiable cause is found, which is termed idiopathic infertility. Idiopathic infertility is defined as infertility without a clear, identifiable cause despite thorough evaluation. Emerging research suggests that oxidative stress and environmental factors play a significant role in idiopathic male infertility [2]. When the underlying cause is identified, pharmaceutical agents can be administered to address the issue. However, up to 25% of male infertility cases remain idiopathic, where the cause is unknown [3].

The European Association of Urology (EAU)’s Male Sexual and Reproductive Health Guidelines strongly recommend drug treatment for the pathophysiology of hypogonadotropic hypogonadism. However, the evidence for the drug treatment of idiopathic male infertility remains unclear [4]. Testicular endocrine and exocrine function, including testosterone biosynthesis and spermatogenesis, are tightly regulated by the hypothalamic–pituitary–gonadal (HPG) axis. High intratesticular testosterone levels and the stimulation of Sertoli cells by follicle-stimulating hormone (FSH) are necessary for spermatogenesis [5]. Although testosterone’s effect on the primary or secondary spermatocyte is not crucial for the completion of male meiosis, its influence on Sertoli cells is vital for the process.

Despite their importance in spermatogenesis, high doses of testosterone and other androgens exhibit contraceptive properties by creating a negative feedback loop in the HPG axis. This feedback blocks the stimulation of intratesticular testosterone production by luteinizing hormone (LH) and the stimulation of Sertoli cells by FSH. Maintaining the reproductive axis to increase the intratesticular testosterone levels in the testes is the primary therapeutic goal for most identified causes of male infertility. However, no specific medical treatment has been found for some men with primary testicular failure or idiopathic male infertility, and empirical treatments are often employed. Most currently available empirical therapies target the HPG axis, and new treatment options are rapidly emerging in this area, offering hope in addressing male infertility.

Idiopathic male infertility is often associated with increased oxidative stress. Thus, it is important to evaluate how antioxidant intake affects sperm. Many previous studies have been inadequately designed or lacked proper controls. This article provides a narrative review of the current empirical treatments for male infertility and assesses the evidence for their effectiveness. The aim is to address research gaps and offer a clearer understanding of the potential benefits and limitations of these treatments.

## 2. Literature Search and Review Approach

### 2.1. Literature Search Strategy

This narrative review is based on a comprehensive literature search conducted across several databases, including PubMed, Web of Science, Scopus, and Google Scholar. The search focused on studies published between January 1979 and May 2024. The keywords used in the search included “male infertility”, “empirical therapies”, “sperm quality”, “oxidative stress”, “antioxidants”, “lifestyle changes”, “weight loss”, “physical activity”, “smoking cessation”, “alcohol consumption”, “aromatase inhibitors”, “selective estrogen receptor modulators”, “gonadotropins”, “phosphodiesterase-5 inhibitors”, “dopamine agonists”, “kallikrein”, “indomethacin”, “low-dose corticosteroids”, “alpha-blockers”, and “nitric oxide donors”.

### 2.2. Inclusion and Exclusion Criteria

To ensure the inclusion of relevant and high-quality studies, the following criteria were applied.

Inclusion Criteria:

- Peer-reviewed articles published within the period of 1979 to 2024;
- Studies that explore the pathophysiology and treatment of male infertility, particularly idiopathic infertility;
- Research examining the role of oxidative stress, environmental factors, and lifestyle modifications in male infertility;
- Articles discussing potential treatments for male infertility, particularly those involving lifestyle modifications and empirical therapies;
- Systematic reviews, meta-analyses, clinical trials, and comprehensive reviews related to male infertility and its treatment.

Exclusion Criteria:

- Publications not in English;
- Studies that do not directly address male infertility or are not relevant to the empirical treatment approaches discussed in the review;
- Articles without accessible full texts;
- Publications predating 1979, except where they provide foundational knowledge.

### 2.3. Data Extraction and Synthesis

For this narrative review, data extraction was performed independently by two reviewers, who focused on essential study elements such as the design, sample size, population characteristics, main findings, and conclusions. In cases of discrepancies, a third reviewer was consulted to reach a consensus. The synthesis involved integrating findings from various studies to provide an overview of the current understanding regarding male infertility and its empirical treatment, highlighting common themes, clinical implications, and potential treatment avenues. This approach aimed to identify both consensus and divergent findings across studies, providing a nuanced understanding of the field.

### 2.4. Quality Assessment and Limitations

While this narrative review does not include a formal quality assessment as in systematic reviews, an emphasis was placed on including studies with rigorous methodologies and relevance to the review’s topic. Potential limitations of this review include the narrative approach, which may not capture the full scope of the literature; possible publication bias; and the inclusion of studies with smaller sample sizes. These limitations may affect the generalizability of the findings. Future research should aim to overcome these limitations through larger, more diverse studies, particularly in the context of oxidative stress and empirical therapies for male infertility.

## 3. Improving Male Fertility through Healthy Lifestyle Habits

Idiopathic infertility may be caused by environmental and lifestyle variables that may synergize with genetic susceptibility, as shown by studies [6,7]. Therefore, lifestyle modification may improve sperm parameters.

### 3.1. Weight Loss

Obesity has long been linked to male infertility due to its detrimental effect on sperm quality and reproductive hormones. Excess body fat increases the levels of oxidative stress and inflammation, which impair spermatogenesis. This leads to a decrease in sperm counts, motility, and morphology. Additionally, excess body fat contributes to hormonal imbalances, including elevated estrogen levels and reduced testosterone production. These factors together are detrimental to male fertility [8].

Several studies have indicated that weight loss through dietary changes and increased physical activity can significantly improve sperm parameters [6,8,9]. The mechanisms behind these improvements are multifaceted. Weight loss helps to lower systemic inflammation, reduce oxidative stress, and restore reproductive hormone balance. Specifically, weight loss can raise testosterone levels while simultaneously decreasing estrogen levels, directly benefitting spermatogenesis. Additionally, weight loss reduces insulin resistance, which has also been associated with impaired fertility [9].

However, it is important to note that data obtained from randomized controlled trials (RCTs) are inconclusive. A recent meta-analysis of 28 cohort studies with 1022 participants found that sperm quality and function did not improve in morbidly obese men after bariatric surgery [10]. There are limited data on the efficacy of assisted reproductive technology (ART). However, weight reduction may help with secondary hypogonadism caused by obesity. This improvement may lead to better outcomes for couples seeking infertility treatment. Additionally, weight loss is critical for the overall well-being of the male partner [9,10].

Overall, weight loss remains a key recommendation for men with obesity-related infertility. Weight loss not only improves sperm parameters but also contributes to greater overall health benefits and lowers the risk of comorbidities that further compromise fertility. Long-term studies are still needed to fully elucidate the extent to which weight loss can reverse infertility in men with obesity, particularly those with severe forms of the condition.

### 3.2. Physical Activity

The World Health Organization (WHO) recommends regular physical activity to reduce the risk of chronic systemic diseases [11]. In a recent meta-analysis, high-intensity (40–80 metabolic equivalents of tasks (METs)/week) or even moderate-intensity (20–40 METs) physical activity was found to positively influence semen parameters, particularly the sperm concentration and progressive motility [12]. However, engaging in high-level physical activity (>80 METs/week) might potentially have negative impacts on these factors, including progressive motility. Moreover, some activities, such as cycling, have been linked to decreased sperm concentrations due to physical exertion and elevated temperatures in the pelvic area. These results indicate that the intensity and type of physical exercise play a crucial role in determining its effect on male reproductive health [12].

In addition, moderate physical activity also promotes hormonal balance, leading to increased testosterone levels and improved spermatogenesis, effects that are comparable to those seen with weight loss [9].

In conclusion, while moderate physical activity supports fertility, high-level physical activity, as well as specific activities like cycling, may pose risks. Therefore, maintaining a balanced approach to exercise is key to optimizing fertility outcomes in men.

### 3.3. Smoking

According to epidemiological statistics, one in three men of reproductive age smokes, with Europe showing the highest smoking rates among all WHO regions [13]. A significant meta-analysis of 20 studies involving 5865 individuals has shown that smoking correlates unfavorably with sperm parameters [13]. Specifically, smoking has been linked to reduced sperm concentrations, motility, and morphology. It also increases sperm DNA fragmentation and oxidative stress. The toxins in cigarette smoke, such as nicotine, cadmium, and reactive oxygen species, impair spermatogenesis and negatively affect the overall quality of sperm [14,15].

Previous experiments by our laboratory in rats have shown that nicotine has a dose-dependent negative effect on sperm that can be reversed by smoking cessation [16]. The negative effects of smoking are not limited to sperm quality but also extend to the hormonal balance, with smokers often exhibiting lower testosterone levels and higher estrogen levels, both of which are detrimental to fertility. However, smoking cessation has been shown to reverse many of these effects [17].

A case study demonstrated an improvement in sperm parameters after a three-month smoking cessation program [18]. In addition to improvements in sperm motility and morphology, smoking cessation also leads to a reduction in oxidative stress markers, allowing for better sperm DNA integrity [19]. Sperm production and quality begin to recover as early as three months after cessation, with more significant improvements seen over extended periods of abstinence from smoking [20].

Similar results were recently published in an uncontrolled study suggesting that ART is more effective when the male partner stops smoking [21]. Couples undergoing ART treatment showed higher pregnancy rates and better outcomes when the male partner had quit smoking prior to treatment, further supporting the recommendation for smoking cessation as a critical intervention in the treatment of male infertility [22,23].

In conclusion, smoking has a well-documented negative impact on male fertility, but the effects are reversible with smoking cessation. Quitting smoking should be a primary recommendation for all men seeking to improve their fertility outcomes, particularly those undergoing ART.

### 3.4. Alcohol Consumption

According to data from a recent meta-analysis that included 15 cross-sectional studies involving 16,395 men, moderate alcohol consumption showed no adverse effects on sperm parameters. In contrast, excessive alcohol consumption may affect male fertility [24]. Chronic high alcohol consumption (>two drinks/day) may decrease testosterone levels and increase estrogen, negatively affecting spermatogenesis [25]. Prolonged heavy drinking also contributes to oxidative stress, leading to sperm DNA damage, which can impair fertilization and embryo development, increasing the risk of infertility and miscarriage [26,27].

Fortunately, studies have shown that abstinence from alcohol for several months can restore normal hormone levels and improve sperm quality, typically within three to six months [28,29]. Improvements include increases in sperm count, motility, and morphology, demonstrating that the detrimental effects of alcohol on male fertility can often be reversed through lifestyle changes [24].

**Figure 1:**
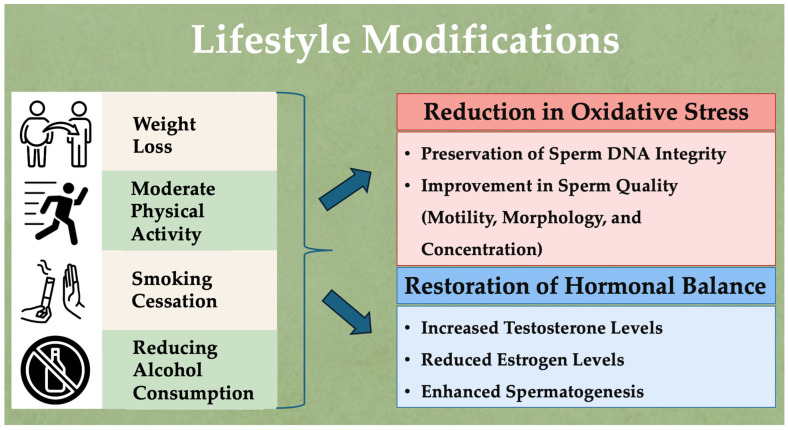
Lifestyle modifications to reduce oxidative stress, restore hormonal balance, and enhance male fertility.

Figure 1 illustrates the key lifestyle modifications that can reduce oxidative stress, restore hormonal balance, and improve sperm parameters, thereby enhancing male fertility.

## 4. Empirical Treatments for Male Infertility with Medicines

The empirical treatments for male infertility can be classified based on their primary therapeutic goals: stimulating spermatogenesis, improving sperm quality, enhancing sexual function, and managing specific pathological conditions.

### 4.1. Stimulating Spermatogenesis

These treatments focus on directly increasing the production of sperm by stimulating hormonal pathways or enhancing testicular function.

#### 4.1.1. Gonadotropins

Gonadotropins are hormones that play a crucial role in regulating spermatogenesis and testosterone production in males. The primary gonadotropins used in the treatment of male infertility are human chorionic gonadotropin (hCG) and human menopausal gonadotropin (hMG). hCG acts similarly to LH, stimulating the Leydig cells in the testes to produce testosterone. This increase in testosterone is crucial in initiating and maintaining spermatogenesis. hCG is particularly useful in treating hypogonadotropic hypogonadism, where the body does not produce enough gonadotropins [30]. hMG, which contains both LH and FSH, is used to stimulate the Sertoli cells in the testes directly. FSH is essential for spermatogonial proliferation and maturation, while LH stimulates testosterone production. The combination of these hormones in hMG makes it particularly effective in treating men with primary testicular failure, where the testes are unresponsive to endogenous gonadotropins [30].

Men with hypogonadotropic hypogonadism, who are unable to secrete their own gonadotropins, may benefit from gonadotropins such as hCG, FSH, or hMG. Treatment is based on the induction of spermatogenesis by drug therapy (hCG, hMG, recombinant FSH, highly purified FSH) [31]. A meta-analysis suggests that combining hCG and FSH is the optimal treatment strategy [32]. Recombinant hCG, LH (rLH), or FSH (rFSH) does not appear to have advantages over urine-derived formulations [32]. However, less is known about how beneficial they are, alone or in combination, in treating normogonadotropic oligospermia [33]. The effects of FSH stimulation alone have been investigated in two randomized controlled trials, one using pure hMG [34] and the other using recombinant human FSH [35]. While one study claimed a benefit of FSH for a specific population in a post hoc analysis, other studies found no improvement in pregnancy rates with FSH [34]. Foresta et al. investigated the effects of rFSH with hCG on sperm parameters and pregnancy rates in severe testiculopathies after high plasma FSH concentrations were suppressed. When endogenous high plasma FSH levels are lowered, as in this randomized controlled trial in men with severe male infertility, sperm parameters improve [36].

Although hCG has been the subject of several uncontrolled studies, the efficacy of hMG in treating idiopathic oligospermia in men has been investigated in only a few studies [33]. The results of these studies were mixed [33], and the only randomized, double-blind, placebo-controlled crossover study of the treatment of normogonadotropic men with idiopathic oligoasthenoteratospermia with hCG/hMG showed no improvement in semen parameters or pregnancy rates [37]. Acne and changes in libido are two of the less serious side effects of an otherwise risk-free drug. Because of the high cost and the lack of convincing results from controlled trials, this drug is not usually recommended in men without proven hormonal imbalances. The pregnancy rates after gonadotropin treatment in a carefully selected subpopulation of infertile men, perhaps in conjunction with ART, require further investigation [38].

Recent studies on gonadotropins in male infertility include a meta-analysis evaluating the effect of FSH in normogonadotropic patients with idiopathic oligozoospermia [39]. The studies were divided into three groups based on their FSH dose: low (175–262.5 IU weekly), medium (350–525 IU weekly), or high (700–1050 IU weekly). According to the meta-analysis, high FSH doses were found to increase the sperm concentration, total sperm count, and progressive motility. Another study investigated FSH treatment in men with azoospermia due to spermatogenic dysfunction. This prospective case–control study examined 40 men who had previously failed microscopic testicular sperm extraction (mTESE) [40]. The participants were divided into two groups: one received three months of FSH treatment, while the control group received no treatment. A second mTESE was performed on the patients, and it was found that two of 20 patients in the treatment group had positive sperm, but this was absent in the control group. Although this was not statistically significant, this observation is worth studying in a large experiment before being translated into clinical practice. A recent retrospective study by Ozman et al. reported an 18.4% success rate after repeated mTESE without medical assistance, indicating ongoing uncertainty in managing these cases [41]. This raises doubts about whether the results of Verdi et al. would be applicable to a larger population of men [40].

Clarifying the differences in efficacy between various forms of gonadotropins is essential. hCG is primarily used to boost testosterone production in men with hypogonadotropic hypogonadism, whereas hMG, which contains both LH and FSH, is more effective in cases requiring the direct stimulation of spermatogenesis. Combination therapy with hCG and hMG is often used for men with more severe infertility issues to maximize the efficacy [38].

In summary, while hCG is effective in boosting testosterone levels, hMG provides a comprehensive approach to stimulating spermatogenesis. The combination of both therapies is often necessary for the treatment of severe infertility cases, and further studies are required to optimize the treatment protocols and improve pregnancy outcomes.

#### 4.1.2. Selective Estrogen Receptor Modulators

Selective estrogen receptor modulators (SERMs) function by blocking estrogen’s negative feedback in the hypothalamus and pituitary gland, which leads to an increase in the secretion of gonadotropin-releasing hormone (GnRH) and subsequently pituitary gonadotropins. This hormonal cascade stimulates spermatogenesis, making SERMs a valuable off-label treatment option for hypogonadal male infertility [42]. Clomiphene citrate and tamoxifen are two widely used SERMs in this context, with clomiphene being particularly noted for its role in binding to estrogen receptors in the hypothalamus. By preventing estrogen from exerting its negative feedback effect, clomiphene increases GnRH secretion, thereby boosting the release of gonadotropins (LH and FSH) from the pituitary gland and enhancing spermatogenesis [43]. Despite their potential, the efficacy of SERM therapy in ART cycles is not well established. According to the American Urological Association, the benefits of SERM therapy in ART cycles are limited, with inconsistent outcomes reported across different studies [44].

A recent retrospective study explored the threshold testosterone level for effective SERM treatment, hypothesizing that hypogonadal men with idiopathic infertility and a total serum testosterone level of 265 ng/dL or less would benefit more from SERM treatment than those with higher baseline testosterone levels [45]. The study demonstrated that, regardless of the baseline blood testosterone level, all 83 men with unexplained infertility who took 50 mg of clomiphene daily for 90 days experienced significant increases in both testosterone and sperm concentrations. This finding suggests that SERM treatment may be beneficial across a range of testosterone levels, supporting the Endocrine Society’s recommendation to consider clomiphene for all patients with idiopathic male infertility who fall below a testosterone threshold of 400 ng/dL [45].

However, clomiphene treatment is not without risks. A systematic review of 384 men from 11 different studies revealed that up to 17% of patients treated with clomiphene citrate experienced a paradoxical decline in sperm parameters, which was not reversible even after the discontinuation of the drug. This unexpected outcome highlights the need for careful patient monitoring and further research into the mechanisms underlying this effect [46].

The overall efficacy of SERMs in increasing pregnancy rates remains contentious. An initial meta-analysis of 11 RCTs, where only five were placebo-controlled, concluded that SERMs did not significantly increase the pregnancy rate in the 459 patients studied [47]. This conclusion was supported by a subsequent Cochrane review, which, despite noting positive effects on hormonal markers, failed to confirm these results in terms of pregnancy outcomes. More recently, Chua et al. performed a meta-analysis of data from 11 RCTs, and their results showed that SERMs were associated with significantly increased pregnancy rates [48]. Improvements in sperm quality and positive changes in hormonal markers were noted. However, the most recent systematic review indicated that these results were not consistently reproducible. The variability in the findings may be attributed to several factors, including differences in the study design, patient selection criteria, and treatment protocols [42].

The conflicting evidence can be attributed to several factors. Differences in the study design, such as the sample sizes, treatment durations, and patient selection criteria, contribute to inconsistent findings. Variability in patient populations, including differences in their baseline testosterone levels, age, and underlying causes of infertility, can also affect the outcomes. Additionally, discrepancies in how the outcomes are measured and reported, including differences in semen analysis techniques and hormonal assays, further contribute to the mixed results observed across studies.

In summary, while SERMs like clomiphene citrate show promise in treating male infertility by improving hormonal profiles and sperm parameters, the evidence remains mixed. More rigorously designed studies with standardized protocols are necessary to resolve these conflicting findings and establish clear guidelines for the use of SERMs in male infertility treatment.

#### 4.1.3. Aromatase Inhibitors

Aromatase is a cytochrome P450 enzyme found in various tissue types, including the testes, prostate, brain, bone, and adipose tissue. This enzyme is responsible for converting testosterone and androstenedione into estrogens, such as estradiol and estrone, which exert negative feedback in the HPG axis. This feedback mechanism reduces gonadotropin secretion, ultimately impairing spermatogenesis, which is crucial for male infertility. Aromatase inhibitors (AIs), such as anastrozole, function by blocking the activity of aromatase, thereby preventing the conversion of testosterone to estradiol in various tissue types. By inhibiting this conversion, AIs increase the intratesticular testosterone levels, which is essential in stimulating spermatogenesis and improving sperm parameters [49].

Current research has identified low blood testosterone levels and a low testosterone–estradiol ratio, often linked with increased aromatase activity, as significant contributors to male infertility. Aromatase inhibitors, therefore, have been increasingly used off-label to treat infertility by enhancing endogenous testosterone production and promoting spermatogenesis [50]. Both steroidal (e.g., testolactone) and non-steroidal (e.g., anastrozole, letrozole) aromatase inhibitors have demonstrated efficacy in improving sperm quality, although their use remains off-label, without Food and Drug Administration (FDA) approval [50].

A recent retrospective study indicated significant improvements in hormone profiles and sperm characteristics in men treated with anastrozole, especially among those with a body mass index (BMI) of 25 or more who were diagnosed as hypogonadal or subfertile. After five months of treatment, these men showed significant improvements in their hormone profiles and sperm characteristics, with a clinical pregnancy rate of 46% [51]. However, it is crucial to note that these findings, while promising, are based on a retrospective analysis. Therefore, more robust, prospective RCTs are necessary to definitively establish the efficacy and safety of aromatase inhibitors in treating idiopathic male infertility.

The current literature on AIs in male infertility, though encouraging, is limited by small sample sizes and a lack of long-term studies. This highlights the necessity for more comprehensive research to establish clear guidelines and to explore the differential effects of various types of AIs. Future studies should focus on identifying the specific patient populations that would benefit most from these treatments, determining the optimal dosing regimens, and assessing the long-term safety outcomes [50].

### 4.2. Improving Sperm Quality

These treatments are aimed at enhancing the quality of sperm by reducing oxidative stress and addressing inflammation, thus improving overall sperm function and viability.

#### 4.2.1. Antioxidant and Micronutrient Supplements

Idiopathic infertility is thought to be caused by oxidative stress, considered one of the essential components in the pathophysiology of idiopathic infertility. The end products of oxidative stress, reactive oxygen species, can potentially impair sperm function by acting at multiple levels. These levels include the lipid peroxidation of the plasma membrane, which can impair sperm motility, the acrosome response, and chromatin maturation, leading to increased DNA fragmentation [52]. As a result, the sperm concentration from reactive oxygen species (ROS) is negatively related to ART outcomes [53]. Because it has long been suspected that ROS and oxidative stress contribute to idiopathic male infertility or subfertility, antioxidant therapy has emerged as a promising avenue for treatment. However, despite widespread interest, the clinical application of antioxidants remains controversial due to inconsistent findings and methodological limitations in existing studies.

Numerous antioxidants, including vitamin C, coenzyme Q10 (CoQ10), L-carnitine, and glutathione, have been investigated for their potential to improve sperm quality. Definitive consensus statements on the use of antioxidants in idiopathic male infertility are not yet possible, and this is often attributed to the high risk of bias due to underreporting of randomization, a lack of follow-up on live birth and pregnancy rates, high patient turnover, and small overall sample sizes [54,55].

The literature presents conflicting findings on the efficacy of antioxidant therapy. For example, a Cochrane systematic review and meta-analysis by Agarwal et al. showed that antioxidant therapy had a positive effect on the number of live births and pregnancies in subfertile couples undergoing ART. This review, which included 34 RCTs and 2876 couples, suggested that antioxidant supplementation could be beneficial [56]. Researchers found results comparable to those previously published in the most recent meta-analysis, which included 61 trials and 6264 infertile men aged 18 to 65 [57]. Similar results were obtained in a recent prospective study investigating the effects of lifestyle changes and oral antioxidants (containing a combination of multivitamins, CoQ, omega-3, and oligo-elements) on semen parameters over three months [58]. Ninety-three patients with a history of IVF or intracytoplasmic sperm injection (ICSI) attempts and ten healthy male controls participated in this study. No significant changes in sperm parameters or static oxidation–reduction potential were observed between the treatment and control groups.

In contrast, the Males, Antioxidants, and Infertility (MOXI) study, a more recent and robust multicenter, double-blind RCT, attempted to address the problem of insufficient power in studies. A total of 174 male participants were evenly divided into two groups: those who received a commercially available combination of vitamins C, E, selenium, L-carnitine, zinc, folic acid, and lycopene (*n* = 85) and those who received a placebo (*n* = 86). Live birth was the primary endpoint, whereas pregnancy within six months of therapy was secondary. After three months of treatment, an internal pilot study used the sperm parameters and DNA fragmentation index as primary endpoints. The MOXI study found that antioxidants did not improve the sperm parameters or DNA integrity in infertile men compared with a placebo. In addition, after six months, there was no significant difference in the cumulative live birth rate between the antioxidant and placebo groups (15% versus 24%) [59]. A later study on the role of antioxidants in sperm parameters and the birth rate found no significant effect of the blood’s baseline vitamin E, zinc, or selenium levels [60].

The conflicting results between studies like that of Agarwal et al. and the MOXI study can be attributed to several factors. First, differences in the study design, such as the sample sizes, treatment durations, and patient selection criteria, can significantly impact outcomes. Second, variability in the baseline characteristics of the participants, including the underlying causes of infertility and the severity of oxidative stress, may lead to inconsistent responses to antioxidant therapy. Additionally, discrepancies in the antioxidant formulations used, dosages, and combinations of supplements further contribute to the mixed results. The heterogeneity in how outcomes are measured, ranging from sperm parameters to live birth rates, also complicates the comparison of findings across studies.

In addition to the fact that there is no conclusive evidence of a therapeutic benefit, administering antioxidants remains a significant challenge [61]. Few studies demonstrate consistent benefits from antioxidant treatment, and the data are primarily controversial, as shown by extensive reviews such as that of Avila et al. [62]. In addition, Dimitriadis et al. acknowledge that the over-the-counter use of such preparations can be harmful [63]. Moreover, relevant analyses have been performed only on small cohorts, suggesting that the available evidence is of low quality [62]. For this reason, antioxidants are not currently recommended by the European Guidelines on Sexual and Reproductive Health for men with infertility. As reported by Symeonidis et al., the main target of antioxidant therapy does not affect sperm quality [64].

In the last two years, eight RCTs have been published on this topic; six were placebo-controlled. Although some combinations showed improvements in sperm parameters, the differences were generally minor, and none of the studies showed a significant difference in pregnancy rates because they were small studies. Coenzyme Q10 (CoQ10) [65,66], L-carnitine, N-acetylcysteine (NAC) [67,68,69,70,71], and vitamin D [72] are examples of combinations of antioxidants that have been shown to improve sperm parameters.

However, each of the above studies had many significant limitations. The data were from poor-quality RCTs, with a severe risk of bias due to inadequate methods of reporting randomization. There were also missing data on clinical outcomes, such as the live birth and clinical pregnancy rates. Additionally, the studies had high attrition rates and imprecision, often due to low event rates and small sample sizes [57]. It has not been possible to provide conclusive evidence on which antioxidants or therapeutic regimens should be used to improve sperm parameters and pregnancy rates [57].

In conclusion, while antioxidant therapy shows potential benefits in improving sperm quality, the current evidence is inconsistent and limited by various methodological flaws. There is a clear need for more rigorously designed, large-scale RCTs with standardized protocols and clinically relevant endpoints to establish the efficacy and optimal use of antioxidant therapy in treating male infertility. Until such evidence is available, antioxidant therapy should be approached with caution, particularly given the potential for over-the-counter misuse and the lack of strong clinical guidelines.

#### 4.2.2. Low-Dose Corticosteroids

The use of low-dose corticosteroids in treating idiopathic infertility has attracted significant interest due to their immunomodulatory effects and potential to improve reproductive outcomes. These corticosteroids, including prednisolone, are primarily valued for their ability to suppress chronic inflammatory infiltrates, which are often identified in testicular biopsies and are believed to contribute to infertility through mechanisms such as the production of antisperm antibodies (ASA). ASA can impair sperm function and reduce fertilization rates [73,74].

Corticosteroids are thought to improve fertility by reducing inflammation and modulating the immune response, which might otherwise hinder sperm function or embryo implantation. For instance, in ASA-positive men, prednisolone has been shown to improve fertilization rates and overall IVF outcomes, suggesting that corticosteroids can mitigate the detrimental effects of these antibodies on sperm [75]. Moreover, the anti-inflammatory properties of corticosteroids may counteract chronic inflammation, often exacerbated by autoimmune conditions, which can negatively affect male reproductive health [76].

However, the efficacy of corticosteroids in treating idiopathic male infertility remains controversial. While some studies suggest potential benefits, others indicate that corticosteroids may not significantly improve fertility outcomes in the absence of ASA [77,78]. For example, a systematic review highlighted that corticosteroid treatment did not reduce sperm-bound antibodies or improve IVF outcomes in men with ASA [77]. Additionally, the long-term use of corticosteroids is associated with adverse effects, including hormonal imbalances and potential impacts on testosterone levels, which could further complicate fertility [78].

Moreover, the safety profile of corticosteroids in this context is critical. While they can provide short-term benefits, the risk of side effects, such as immunosuppression and hormonal disruptions, necessitates careful consideration [79]. A balance between the potential benefits and risks of corticosteroid therapy is essential, particularly in light of the alternative treatments available for male infertility, such as ART [79,80].

In conclusion, while low-dose corticosteroids may offer some therapeutic advantages in managing idiopathic male infertility, particularly in cases involving antisperm antibodies, their overall effectiveness and safety profile require further investigation. Clinicians should weigh the potential benefits against the risks of long-term corticosteroid use and consider integrating other treatment modalities to optimize the fertility outcomes.

### 4.3. Enhancing Sexual Function

These treatments focus on improving sexual function, which can directly or indirectly impact fertility by addressing issues such as erectile dysfunction and sperm motility.

#### 4.3.1. Phosphodiesterase-5 Inhibitors (Selective)

Phosphodiesterase-5 inhibitors (PDE5is) are the treatment of choice for erectile dysfunction. However, emerging research suggests that these inhibitors may also have beneficial effects on male fertility by improving semen parameters. A review by Sofikitis et al. explored the potential of PDE5is as an adjuvant therapy in treating male infertility, revealing that these inhibitors may enhance the secretory activity of Leydig and Sertoli cells, which are crucial for testosterone production and spermatogenesis [81]. One of the key mechanisms through which PDE5is may exert their effects is by modulating contractility in the tunica albuginea of the testes and epididymis. This modulation can improve blood flow and support the transport of sperm, potentially enhancing sperm quality. Additionally, PDE5is have been associated with increased prostate secretory function, which may further contribute to improved sperm motility and overall semen quality [81].

Another area of interest is the role of PDE5is in sperm capacitation, a critical process that sperm undergo to gain the ability to fertilize an egg. Some studies suggest that PDE5is may play an essential role in regulating this process, which is vital for successful fertilization. These findings indicate that PDE5is could have broader applications in reproductive medicine beyond their traditional use in treating erectile dysfunction [82].

A recent meta-analysis of nine RCTs involving 1211 participants from various countries has provided more robust evidence of the impact of PDE5is on sperm parameters [83]. In this study, participants received different doses of PDE5is, and their sperm parameters were assessed before and after treatment. The results demonstrated significant improvements in sperm concentrations, motility, and morphology among men treated with PDE5is compared to those receiving a placebo [83].

Despite these promising findings, several limitations need to be addressed. The majority of earlier RCTs on PDE5is were conducted on a smaller scale, often without considering demographic factors such as age, lifestyle, or underlying health conditions, which could introduce bias into the results. Moreover, the heterogeneity of the study designs and patient populations complicates the interpretation and generalization of the findings. These limitations underscore the necessity for larger, placebo-controlled, blinded, randomized trials that account for potential confounding factors and demographic variability.

Furthermore, while the evidence points towards a potential role for PDE5is in enhancing male fertility, the exact mechanisms remain incompletely understood. There is a need for more in-depth research to elucidate how PDE5is influence spermatogenesis, sperm capacitation, and other aspects of male reproductive health. Such studies could pave the way for the development of more targeted therapies that utilize PDE5is in conjunction with other treatments to optimize fertility outcomes.

In conclusion, while PDE5is show potential as an adjunct treatment for male infertility by improving semen parameters, their application in this context is still in its early stages. Future research should focus on addressing the limitations of existing studies, exploring the underlying mechanisms, and conducting large-scale, well-designed clinical trials to establish clear guidelines for their use in male infertility treatment. These efforts are crucial in determining whether PDE5is can be effectively integrated into broader fertility treatment protocols.

#### 4.3.2. Phosphodiesterase-5 Inhibitors (Non-Selective)

Non-selective phosphodiesterase-5 inhibitors, such as pentoxifylline and other methylxanthines, are known to enhance sperm motility in vitro, leading to higher fertilization rates during ART. These agents work by increasing the cyclic adenosine monophosphate (cAMP) levels within sperm cells, improving motility [84].

However, the effects observed in vitro do not consistently translate to systemic administration. When administered systemically, these drugs may have reduced potency due to dilution and broader distribution within the body, affecting various tissue types and potentially leading to off-target effects [85].

Therefore, while non-selective PDE5is may be useful in specific ART protocols, particularly in sperm preparation for procedures like IUI or IVF, their role as standalone treatments for male infertility remains limited. Further research is needed to refine their application in fertility treatments and explore how they might be combined with other therapies to address the root causes of infertility more effectively.

#### 4.3.3. Nitric Oxide Donors

Many physiological processes, such as angiogenesis, growth, puberty, and senescence, are mediated by nitric oxide (NO), a reactive nitrogen species. NO plays an essential role in normal reproduction by regulating steroidogenesis, gametogenesis, and apoptosis in germ cells. NO is critical for male sexual function, including steroidogenesis, erectile function, sperm viability, and the acrosome response. In addition, NO contributes to the maintenance of a stable blood–testis barrier and regulates the communication between Sertoli and germ cells. Increased activity of nitric oxide synthase (NOS) in pathological situations, such as infections, leads to an excess of NO, a proinflammatory mediator that triggers oxidative stress (OS), which negatively affects erectile function and reproduction [86,87].

Modulating defenses and cellular immunity is one of the main functions of L-arginine. It also plays a crucial role in the sperm maturation process. A deficiency in L-arginine leads to impaired sperm metabolism, resulting in impaired motility. L-arginine has been shown to increase sperm counts and motility in individuals with oligospermia and asthenospermia without adverse effects. Under in vitro conditions, L-arginine has been shown to stimulate sperm motility in humans, rabbits, and goats [88].

In conclusion, while NO donors and L-arginine hold promise in enhancing male fertility, their use must be carefully tailored to individual patients’ profiles. A deeper understanding of the dual role of NO in both supporting and potentially impairing reproductive functions is crucial in optimizing treatment strategies and improving clinical outcomes in male infertility.

### 4.4. Managing Specific Pathological Conditions

These treatments are designed to address particular medical conditions that can contribute to infertility, such as hormonal imbalances or specific enzymatic dysfunctions.

#### 4.4.1. Dopamine Agonists

Prolactin-secreting pituitary adenoma is the cause of infertility and hyperprolactinemia. Hypogonadism and male infertility occur when pulsatile GnRH production is suppressed due to elevated prolactin levels. Dopamine agonists have been proposed for the treatment of infertility and pituitary tumors. In the past, bromocriptine and cabergoline were used as therapy. However, cabergoline has been shown to normalize the prolactin levels in 70% of bromocriptine-resistant patients and to suppress prolactin production more effectively than bromocriptine [89,90]. Because of its remarkable efficacy in restoring normal prolactin levels and reducing the prolactin-producing tumor size, cabergoline (0.125–1.0 mg twice weekly) is the treatment of choice. Resistance to dopamine agonists is likely in patients who do not achieve normal prolactin levels at the maximum acceptable dose, in whom the tumor size decreases by less than 50%, and in whom fertility cannot be restored. Increasing the dose or switching from bromocriptine to cabergoline is the current standard of care for adenomas resistant to dopamine agonists. However, the treatment of idiopathic oligozoospermia with bromocriptine has not shown significant improvements over a placebo [91].

In conclusion, while dopamine agonists like cabergoline are indispensable in treating hyperprolactinemia-induced infertility, their utility in idiopathic male infertility is minimal. A more nuanced understanding of infertility’s diverse causes is essential in developing effective treatment strategies, and continued investigation into the roles of dopamine agonists and other potential therapies is warranted.

#### 4.4.2. Kallikrein

Proteolytic kallikrein cleaves kininogen to generate locally active kinins (such as bradykinin and kallidin) during the inflammatory response. In vitro studies have shown that the kallikrein–kinin system plays a role in controlling sperm motility. In a double-blind crossover study with placebo controls, Schill found that 600 kU/day of kallikrein improved the sperm parameters and increased the pregnancy rate from 18 to 38 percent in men treated for 60 days [92]. The beneficial effect of kallikrein therapy was also observed in previous studies by our laboratory [93]. However, other researchers have shown that treating sperm with kallikrein impairs their quality [94]. In addition, treatment with kallikrein has been shown to exacerbate pre-existing inflammatory diseases of the epididymis or prostate.

In conclusion, while kallikrein therapy offers a promising avenue to improve sperm parameters and increase pregnancy rates in some men, its use is not without risks. A more nuanced approach is required, considering both the potential benefits and the limitations of kallikrein treatment in the context of male infertility. Continued research is essential to establish clear guidelines for its safe and effective use.

#### 4.4.3. Indomethacin

Prostaglandins decrease sperm motility in vitro and inhibit testicular steroidogenesis and spermatogenesis in vivo. These results suggest a regulatory effect of prostaglandins that is detrimental to testicular and sperm function. This may mean that blocking prostaglandins promotes conception. Treatment with indomethacin or ketoprofen was associated with increased levels of FSH, LH, and testosterone in a controlled study by Barkay and colleagues. At a daily dose of 75 mg of indomethacin, which also increased the sperm concentration and motility, the pregnancy rate was 35% [95]. More studies are necessary to clarify the subpopulation of infertile men who obtain a benefit from indomethacin administration.

In conclusion, while the inhibition of prostaglandins presents a potential strategy for the treatment of male fertility, particularly in cases where elevated prostaglandin activity is suspected, this approach requires further validation. Future studies should focus on identifying the specific conditions under which prostaglandin inhibition is most effective, developing tailored treatment protocols, and ensuring that the benefits outweigh the risks for the patient population in question.

#### 4.4.4. Alpha-Blockers

The investigation of the effects of alpha-blockers on sperm production and sperm parameters in the epididymides of rats revealed that sperm production in the testes of rats and the sperm concentration in the epididymis were increased [96]. Sperm transport and storage in the rat testis and epididymis are impaired by alpha-blockers [97]. Terazosin has been tested to determine if it has a beneficial effect in men with idiopathic oligozoospermia. In a study by Gregoriou et al., terazosin was shown to have a beneficial effect in patients with idiopathic oligozoospermia, particularly on the sperm concentration [98].

In conclusion, while alpha-blockers like terazosin show potential as a treatment for idiopathic oligozoospermia, their role in male infertility treatment remains to be fully elucidated. A more nuanced approach that considers both the benefits and risks of alpha-blocker therapy, alongside further research to clarify their impact on fertility outcomes, is essential in advancing their use in clinical practice.

Table 1 summarizes the various medical treatments for male infertility, detailing their mechanisms of action, efficacy, and key references supporting their use.

### 4.5. Combination Treatments for Male Infertility

Recent advances in treating male infertility highlight the promise of combination therapies to enhance reproductive outcomes. Researchers have observed improvements in sperm parameters and increased pregnancy rates when treating cases of idiopathic infertility with hormonal treatments combined with other therapeutic agents such as antioxidants.

Hormonal and antioxidant combinations: Numerous studies have explored the synergistic benefits of combining hormonal therapies with antioxidants. Tamoxifen citrate, when combined with natural compounds like Tribulus terrestris, Ecklonia bicyclis, and other antioxidants, significantly increased the sperm concentration and motility. This combination led to higher pregnancy rates compared to tamoxifen alone [99]. Astaxanthin and other antioxidants have proven their worth as supplements to conventional treatments to improve both sperm quality and pregnancy rates [100]. Several small, randomized studies have demonstrated that combining anti-estrogen therapies, such as clomiphene, with antioxidants, like L-carnitine or vitamin E, can improve sperm parameters and increase pregnancy rates [101,102]. Additionally, studies involving diverse combinations of antioxidants have shown mixed results. Paradiso Galatioto et al. highlighted the use of commercial preparations containing N-acetylcysteine and multi-vitamins, which enhanced sperm parameters. However, these combinations did not significantly increase spontaneous conception rates [103]. While some studies debate the impact of these combinations on sperm parameters, the use of zinc, vitamins, selenium, and N-acetylcysteine has consistently resulted in increased sperm motility and/or concentrations [55].

Hormonal combination therapy: Tamoxifen citrate with testosterone undecanoate has been extensively researched, with variable outcomes. A 2003 RCT showed significant improvements in sperm counts, motility, morphology, and spontaneous pregnancies among men with idiopathic oligozoospermia [104]. However, multiple studies have indicated that androgens do not consistently enhance pregnancy rates or sperm parameters in men with idiopathic infertility [105,106,107]. Furthermore, exogenous testosterone therapy is known to suppress the hypothalamic–pituitary axis, leading to reduced intratesticular testosterone and impaired spermatogenesis [107,108,109]. However, recent surveys show that many urologists continue to prescribe testosterone treatment for idiopathic infertility cases, although the practices vary considerably among general and fellowship-trained urologists [110].

Emerging combinations: Novel combination therapies are also being created to address specific needs in male infertility treatment. Clomiphene citrate and anastrozole combination therapy has shown modest but significant increases in post-treatment sperm concentrations compared to anastrozole monotherapy alone [111]. Similarly, combinations involving HCG and letrozole have demonstrated substantial improvements in terms of counts, motility, and morphology in obese men who suffer from idiopathic infertility [112].

Combination therapies offer a promising approach to improving male infertility outcomes, especially in idiopathic cases. However, the evidence remains mixed, and further research is needed. Large-scale, randomized controlled trials are crucial to establish protocols and determine the most effective combinations for different patient populations.

## 5. Future Directions and Research

In the realm of empirical therapy for male infertility, various avenues for future research and clinical practice can be identified from the existing literature. Empirical medical treatment has been a subject of interest, particularly in idiopathic male infertility, with a focus on hormonal treatments and antioxidant supplementation [3]. The concept of male oxidative stress infertility (MOSI) has emerged, suggesting a need for evidence-based treatments targeting the underlying causes of this condition [113].

While empirical therapies such as antioxidants, SERMs, and hormones have shown some improvements in sperm parameters, their effectiveness in enhancing pregnancy rates, a crucial outcome measure, remains inconclusive [114]. The integration of genomic, epigenomic, and environmental factors is essential for a comprehensive understanding of male factor infertility and the development of effective treatments [115]. Additionally, personalized treatment strategies rooted in precision medicine principles could be pivotal in addressing infertility associated with microbial dysbiosis [116].

Furthermore, alternative therapies like acupuncture, pharmacopuncture, and herbal remedies have demonstrated clinical efficacy in improving sperm motility and morphology in idiopathic infertile men [117]. The differentiation of human pluripotent stem cells into advanced spermatogenic cells presents a novel approach to understanding the biological mechanisms underlying male factor infertility [118]. Letrozole has also emerged as a potential treatment for male infertility of unknown causes, influencing hormonal profiles and seminal parameters [119].

To address the current gaps in the evidence, future research should prioritize the following areas: first, developing clear and realistic guidelines for the use of SERMs, antioxidants, and hormonal treatments in clinical practice; second, conducting large-scale, well-designed randomized controlled trials to establish the effectiveness of these therapies in improving pregnancy rates; finally, exploring the role of alternative therapies and precision medicine in creating personalized treatment plans for male infertility. A multidisciplinary approach, incorporating advancements in genomic research, stem cell technology, and alternative medicine, is crucial in advancing the treatment landscape and providing clear, evidence-based recommendations for clinical practice.

## 6. Conclusions

The empirical treatments for male infertility reviewed in this manuscript demonstrate significant potential in improving sperm quality and reproductive outcomes. Key findings include the positive impact of lifestyle modifications such as weight loss, physical activity, smoking cessation, and alcohol moderation. Weight reduction, particularly in obese individuals, enhances sperm parameters and overall reproductive health. Regular physical activity improves semen parameters and hormone profiles, while quitting smoking consistently enhances sperm quality, emphasizing the importance of cessation programs. Although moderate alcohol consumption does not adversely affect sperm parameters, excessive intake can decrease testosterone levels and impair fertility, which can be mitigated by abstaining from alcohol.

SERMs, particularly clomiphene, are recognized as effective and safe empirical therapies for idiopathic male infertility, especially in hypogonadal men. However, the absence of large, well-designed RCTs prevents definitive conclusions about their impacts on pregnancy rates. Aromatase inhibitors may benefit men with low testosterone-to-estradiol ratios, improving their sperm parameters and hormonal profiles, yet their application remains off-label and warrants further investigation.

The use of gonadotropins (hCG, hMG) shows promise in specific subpopulations, particularly in men with hypogonadotropic hypogonadism, although their high cost and inconsistent results in normogonadotropic men discourage routine use. PDE5is demonstrate potential in enhancing sperm motility and morphology, although their exact mechanisms in improving male fertility require further study. Administering exogenous testosterone for the purpose of treating male infertility is not recommended since it hampers the production of sperm.

Antioxidants such as vitamins C and E and coenzyme Q10 may mitigate oxidative stress and improve sperm quality, but the evidence is mixed and further large-scale studies are needed to confirm their benefits. Dopamine agonists, particularly cabergoline, are effective in managing hyperprolactinemia-induced infertility, but their role in idiopathic cases is limited. Other treatments, including kallikrein, indomethacin, low-dose corticosteroids, alpha-blockers, and nitric oxide donors, show variable effectiveness depending on the specific pathological condition and should be considered carefully.

Given the mixed evidence, empirical treatments should be individualized, prioritizing those with the strongest evidence base, such as SERMs and aromatase inhibitors, in the appropriate clinical context. Large, well-controlled studies are necessary to establish clear guidelines and optimize the treatment strategies for idiopathic male infertility. Until such data are available, the careful consideration of patient-specific factors remains crucial in therapeutic decision-making.

## Figures and Tables

**Table 1:** Overview of medical treatments for male infertility.

Medical Treatment	Mechanism of Action	Efficacy	Level of Evidence	References
Gonadotropins	Mimic the action of LH and FSH to stimulate Leydig and Sertoli cells in the testes	↑ Testosterone ↑ Spermatogenesis ↑ Sperm concentration ↑ Progressive motility	High	[30,31,32,33,34,35,36,37,38,39,40,41]
SERMs	Block estrogen’s negative feedback in the hypothalamus and pituitary gland, increasing the release of gonadotropins (LH and FSH)	↑ Hormone levels ↑ Sperm parameters ↑ Pregnancy rates	High	[42,43,44,45,46,47,48]
Aromatase Inhibitors	Block conversion of testosterone to estradiol by inhibiting the enzyme aromatase	↑ Testosterone ↑ Sperm parameters ↑ Pregnancy rates	Moderate to High	[49,50,51]
PDE5 Inhibitors	Enhance Leydig and Sertoli cell secretory activity by inhibiting PDE5	↑ Sperm concentration ↑ Motility ↑ Morphology	Moderate	[81,82,83]
Antioxidants	Reduce oxidative stress by neutralizing ROS	↑ Sperm parameters, mixed evidence ↑ Pregnancy rates ↑ Live births	Moderate to Low	[52,54,55,56,57,58,59,60,67,68,69,70,71]
Dopamine Agonists	Normalize prolactin levels by stimulating dopamine receptors in the pituitary gland	Normalized Prolactin ↑ Fertility	Moderate	[89,90,91]
Kallikrein	Cleave kininogen to generate kinins, which are involved in the regulation of sperm motility	↑ Sperm motility ↑ Pregnancy rates	Low	[92,93,94]
Indomethacin	Block prostaglandin synthesis by inhibiting the cyclooxygenase (COX) enzyme	↑ FSH, ↑ LH, ↑ Testosterone, ↑ Motility	Low	[95]
Low-Dose Corticosteroids	Reduce chronic inflammatory infiltrates and immune response	↑ Sperm quality	Low	[73]
Alpha-Blockers	Increase sperm concentration by relaxing smooth muscle in the reproductive tract	↑ Sperm concentration	Low	[96,97,98]
Nitric Oxide Donors	Regulate steroidogenesis and gametogenesis by enhancing nitric oxide availability	↑ Sperm count ↑ Motility	Low	[86,88]

ROS—reactive oxygen species; LH—luteinizing hormone; FSH—follicle-stimulating hormone; PDE5—phosphodiesterase type 5; SERM—selective estrogen receptor modulator. ↑ indicates an increase in the respective parameter.

## Data Availability

Not applicable.
